# Whole-Cell Microbial Bioreporter for Soil Contaminants Detection

**DOI:** 10.3389/fbioe.2021.622994

**Published:** 2021-02-23

**Authors:** Ni Zeng, Yichao Wu, Wenli Chen, Qiaoyun Huang, Peng Cai

**Affiliations:** ^1^State Key Laboratory of Agricultural Microbiology, Huazhong Agricultural University, Wuhan, China; ^2^College of Resources and Environment, Huazhong Agricultural University, Wuhan, China

**Keywords:** microbial bioreporter, environmental monitoring, soil contamination, heavy metal, organic contaminants, biosensor

## Abstract

Anthropogenic activities have released various contaminants into soil that pose a serious threat to the ecosystem and human well-being. Compared to conventional analytical methodologies, microbial cell-based bioreporters are offering a flexible, rapid, and cost-effective strategy to assess the environmental risks. This review aims to summarize the recent progress in the application of bioreporters in soil contamination detection and provide insight into the challenges and current strategies. The biosensing principles and genetic circuit engineering are introduced. Developments of bioreporters to detect and quantify heavy metal and organic contaminants in soil are reviewed. Moreover, future opportunities of whole-cell bioreporters for soil contamination monitoring are discussed.

## Introduction

Soil is a central ecosystem that sustains humans, plants, and animals. However, the increasing industrial and agricultural activities have released various contaminants such as aromatic compounds and heavy metals into soil, which severely deteriorated soil health and sustainability (Bispo et al., 1999; Gu and Chang, 2001; Wu et al., 2018; Bae et al., 2020). The detection and monitoring of soil contaminants are vital for the security of humans and other biota on earth (Lu et al., 2015).

The conventional techniques for soil contaminant analysis include gas chromatography–mass spectrometry (GC-MS), high-performance liquid chromatography (HPLC), atomic absorption spectroscopy (AAS), atomic fluorescence spectroscopy (AFS), inductively coupled plasma mass spectrometry (ICP-MS), and X-ray absorption spectroscopy (Branyikova et al., 2020; Wang et al., 2020). These instrumental-based analyses enable soil contamination measurements with high sensitivity and accuracy. However, the conventional chemical approaches measure chemical availability of soil contaminants rather than the bioavailability (Hou et al., 2014). Based on the definition proposed by the National Research Council’s (NRC) committee and the ISO/TC 190 working group, bioavailability is a dynamic process that comprises multiple steps (NRC, 2003; IOS, 2008). It includes the environmental availability of contaminants in soil, the contaminant uptake by organisms, and accumulation and toxic effects of contaminants within organisms (Harmsen, 2007; Kim et al., 2015). Therefore, a biological approach is required to complement chemical analyses to assess the actual ecotoxicological and health risks.

Whole-cell bioreporter, a promising technique to address this issue, allows a rapid detection of bioavailable contaminants in soil (Liu et al., 2011; Song Y. et al., 2014; Francisco et al., 2019). The bioreporter technology uses microbial cells as molecular recognition elements to sense analytes in the environment and exhibits a measurable signal (Su et al., 2011; Turdean, 2011; Bilal and Iqbal, 2019). Due to the simplicity, portability, better detection limit, rapid response, and cost-effectiveness, bioreporters are ideal approaches to assess the bioavailable fraction of contaminants (Wang X. et al., 2014; Bilal and Iqbal, 2019). This review summarized the current state of whole-cell bioreporter in soil contaminant monitoring. The principles and configurations of whole-cell bioreporters are briefly introduced. The applicability of bioreporters in soil contaminant detection is discussed. The recent advances in the application of bioreporters to assess the contamination status of heavy metals and organic pollutants were surveyed. Future prospects of whole-cell bioreporters are further discussed.

## Whole-Cell Microbial Bioreporter Designs for Contaminant Measurement

**FIGURE 1:**
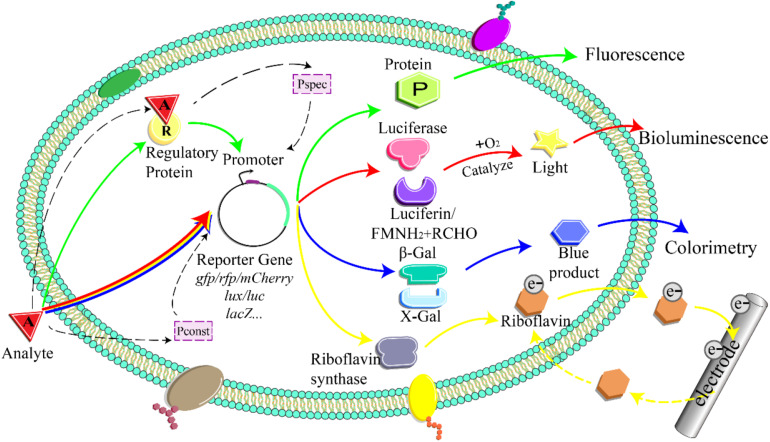
Detection principle of whole-cell microbial bioreporter. The analyte activates or inhibits the expression of the specific promoter and downstream reporter gene. P_spec_, specific promoter; P_const_, constitutive promoter; FMNH_2_, reduced flavin mononucleotide; RCHO, aldehyde; β-Gal, β-galactosidase; X-Gal, 5-bromo-4-chloro-3-indolyl-β-D-galactopyranoside; e^–^, electron.

A whole-cell bioreporter comprises three parts: the sensor, the genetic logic circuit, and the actuator (Figure 1; Kaur et al., 2015; Gupta et al., 2019). The biological sensor element responds to target compounds and triggers a cascade of biological reactions (Bilal and Iqbal, 2019). Genetic logic circuits link the sensors and actuators and convert the bioreaction into a detectable signal proportional to the analyte concentration (Su et al., 2011; Jia et al., 2019).

### Sensor Elements

The specificity and sensitivity of bioreporters are mainly determined by sensor elements (Shin, 2011). Based on the specificity, bioreporters can be categorized into non-specific and specific bioreporters (Jarque et al., 2016). The non-specific bioreporter responds to toxic substances that cause stress (Sorensen et al., 2006). The presence of toxic contaminants suppresses the transcriptional activity of a constitutive promoter to decrease the output signal or triggers the transcription of the reporter genes through stress-responsive promoters. For example, a non-specific bioreporter was deigned *via* fusing the reporter gene to a constitutive promoter P*_*psbA*_* (Elväng et al., 2001; Gu and Chang, 2001). When exposed to toxic chemicals, the expression of the reporter gene is inhibited, which induces a decrease in signal intensity. Another example is a non-specific stress-responsive bioreporter constructed *via* fusing the bioluminescent reporter *luxCDABE* from *Photorhabdus luminescens* with SOS-controlled *recA* promoter in *Acinetobacter baylyi* ADP1 (Sorensen et al., 2006; Song et al., 2009). When exposed to genotoxic contaminants, DNA damage triggered an SOS response to activate *recA* promoter for the expression of *lux* genes (Song Y. et al., 2014).

Specific bioreporters contain a regulatory protein responsible for activating or repressing the promoter activities as the sensing domain (Burmølle et al., 2006; Sorensen et al., 2006). When the target contaminants are present, the regulatory protein binds with the contaminants and undergoes a conformational change, which activates or inhibits its DNA binding and modulates the expression of reporter genes. The proteins from MerR family recognize specific target metal ions, such as the mercury-sensing regulatory protein MerR and lead-sensing regulatory protein PbrR (Brown et al., 2003). For example, mercury-specific bioreporters are constructed *via* fusing reporter genes such as *luxAB* and *gfp* to the downstream of the *merR* promoter (Branyikova et al., 2020).

### Genetic Logic Circuits

Genetic circuits connect sensor apparatuses and actuators that program cells to respond to signal inputs and generate precise behavioral actions (Wang and Buck, 2012; Saltepe et al., 2018). Genetic circuitry is capable of integrating multiple input signals and resulting in specific biochemical behaviors that can enhance the specificity and sensitivity of bioreporters (Lim, 2010). Based on the basal transcriptional activity of promoters, there are two types of non-specific bioreporters (Jiang et al., 2020). The first one comprises a constitutively expressed promoter to maintain a high basal transcriptional activity. When exposed to toxic contaminants, the activity of the promoter is suppressed to decrease the output signal. In the second non-specific bioreporter, the reporter gene is fused with a stress-responsive promoter, whose activation induces the expression of the reporter gene under stress conditions (Burmølle et al., 2006). Specific bioreporters are composed of specific regulatory proteins responsible for promoter activities. The binding of target contaminants with the regulatory protein results in the induction or repression of reporter gene expression (Reyes-Caballero et al., 2011). The regulator proteins are generally continuously expressed to function as repressors that inhibit the expression of reporter genes. When contaminants are present, the repressor protein interacts with the contaminants and activates the reporter gene expression. For example, the regulator *alkR* in the bioreporter sensing alkanes is constitutively transcribed to inhibit the signal output. Its interaction with alkanes activates the promoter P*_*alk*__*M*_* and expression of downstream reporter genes (Zhang et al., 2012).

Most bioreporters were designed to target a single contaminant. To sense multiple contaminants concurrently, logic gates were incorporated in bioreporters. The typical logic gates include amplifier, NOT, AND, and OR (Silva-Rocha and de Lorenzo, 2008; Katz, 2019). When the input signal is present, the amplifier generates one output and the NOT gate suppresses the output. The AND and OR gates are capable of computing multiple inputs and generating one output. For example, a dual-input “AND gate” can respond to two inputs and only be expressed when both inputs are detected, while the OR gate generates output when either one of the inputs is present (Song H. et al., 2014). A triple-input AND logic gated bioreporter system was engineered to detect three different heavy metals by coupling two dual-input AND gates (Wang et al., 2013). The first dual-input AND gate can detect arsenic and mercury *via* two specific inducible promoters (P*_*arsR*_* and P*_*merT*_*). The output of the first bioreporter is the quorum-sensing molecule 3OC_6_HSL. The second AND gate comprises quorum-sensing promoter P*_*luxI*_* and copper-responsive promoter P*_*cusC*_*, which can sense quorum-sensing molecule and copper ion with *rfp* as output readout. Consequently, the AND gated triple-input bioreporter system based on two AND gates can detect and integrate arsenic, mercury, and copper concentrations in parallel.

Genetic logic gates can also function as a biological filter and an amplifier (Kim et al., 2016; Jia et al., 2018, 2019; Saltepe et al., 2018). A positive feedback amplifier using LuxR auto tuning elements improved the sensitivity and specificity of an arsenic whole-cell bioreporter. The arsenic-inducible promoter P*_*ars*_* and its regulatory gene *arsR* were coupled to the reporter transcriptional activator *luxR* in the first plasmid. The *mCherry* gene and *luxR* gene were placed downstream of the promoter P*_*luxI*_* in the second plasmid to form a positive feedback loop. When arsenic ion binds to ArsR to alleviate its inhibition of P*_*ars*_* promoter, the expression of LuxR in the first plasmid is activated, which initiates the expression of *mCherry* and *luxR* in the second plasmid. Its expression product LuxR in the second plasmid activates the expression of its own *mCherry* (Jia et al., 2019). The minimum detection limit was reduced by one order of magnitude. Similarly, a lead whole-cell bioreporter containing positive feedback amplifiers was 15–20 times more sensitive than the one without amplifiers (Jia et al., 2018). Compared with the constitutively expressed regulator, the positive feedback regulator enhances the specificity and sensitivity of bioreporters (Jia et al., 2019). However, the main concern of the positive feedback regulator is the high noise level and the false-positive signal from leaky promoters (Bansal et al., 2010).

### Actuator Elements

Genetic logic circuits respond to the input signal and convert it into a detectable signal output (Figure 1) (e.g., fluorescence, bioluminescence, colorimetry). Reporter genes play a crucial role in the signal transduction process. Optical-based reporters are mostly commonly used in soil contamination detection (Burmølle et al., 2006; Xu et al., 2013). The target analyte induces or represses the expression of the fluorescent and luminescent reporter genes, such as *lux*, *lacZ*, and *gfp* (Shin, 2011). Although the actuators based on visual detection are characterized by simple operation, good stability, high sensitivity, and fast response speed, they come with limitations (Burmølle et al., 2006; Jarque et al., 2016).

The *lacZ* gene of *Escherichia coli* encoding β-galactosidase was one widely used reporter element. The color change during enzyme assay allows the colorimetric detection of β-galactosidase activity. Compared with bacterial luciferase and green fluorescent protein, β-galactosidase can be expressed with low metabolic activities and the colorimetric reaction of β-galactosidase does not require oxygen. Another key strength of the β-galactosidase-based actuator is the high sensitivity. The detection limit of a β-galactosidase-based cadmium bioreporter is 10 pM, which is three orders of magnitude lower than that of a *gfp*-based construct in the same host strain (Shetty et al., 2003). However, the colorimetric assay of β-galactosidase requires disruption of host cells and addition of substrates. Therefore, chromogenic reporter genes like *lacZ* were not frequently employed in soil contamination detection.

Bioluminescent and fluorescent actuators emit light or fluorescence upon soil contaminants without cell disruption and substrate addition. Due the ease of visualization, luminescent and fluorescent reporter genes were preferable than chromogenic reporter genes. The bacterial luciferase encoded by *luxCDABE* operon from *Aliivibrio fischeri* or *luxCDABE* operon from *Photobacterium leiognathi* converts fatty aldehyde to fatty acid coupled with the oxidation of FMNH_2_ and emits a blue light as readout (Sorensen et al., 2006). The luciferase substrate, aldehyde, is subsequently regenerated *via* the multienzyme reductase complex encoded by *luxCDE*. However, as the bacterial luciferase reaction requires oxygen, *lux* reporter genes are not applicable for anaerobic environment. Additionally, it is noteworthy that light output is an energy-demanding process that is dependent on the metabolic state of host cells. Fluorescent proteins, especially green fluorescent protein (GFP) from jellyfish *Aequorea victoria*, are also widely applied in bioreporter design (Daunert et al., 2000). The measurement of GFP fluorescence only requires light excitation. The green fluorescent signal allows single-cell detection *via* microscopy or flow cytometry (Gutierrez et al., 2015; Kaur et al., 2015). The GFP protein also requires the presence of oxygen to fold and fluoresce properly. However, natural GFP takes a long time to fold and is difficult to denature, which may cause false-positive results. Therefore, the *lux*-based actuator is preferred in constructing bioreporters with a fast response time (Valkova et al., 1999; Hernández-Sánchez et al., 2016).

Due to the opacity of the soil matrix, the optical-based bioreporter requires preparation of soil solution or soil extract. The electrochemical bioreporter system featured by minimal sample pretreatment and rapid detection is a promising candidate to improve the measurement. The principles of electrochemical measurement are based on the electrochemical properties of electroactive products catalyzed by reporter enzymes or biological interaction processes, which generate a measurable electrochemical signal. For instance, ampere-based whole-cell electrochemical bioreporters can be constructed *via lacZ* or *phoA* reporter gene (Paitan et al., 2004; Cortes-Salazar et al., 2013). Expression of β-galactosidase or alkaline phosphatase converts p-aminophenyl-β-D-galactopyranoside (PAPG) or p-aminophenol phosphate (PAPP) into p-aminophenol (PAP). PAP is oxidized at the electrode and can be converted to a current signal using the chronoamperometric technique (Paitan et al., 2004). One recent study fuses P*_*zntA*_* promoter with *zntR*, *rib*, and *oprF*, which respond to Zn and trigger riboflavin and porin production in *E. coli*. The production of outer membrane porin and electron shuttles enhanced extracellular electron transfer, which exhibited a linear relationship between the maximum voltage and Zn concentration (Khan et al., 2020). Microbial cells can also function as an adsorbent to immobilize heavy metal ions on bioelectrode. The contaminants can be then measured *via* differential pulse anodic stripping voltammetry analysis (Dali et al., 2018). Moreover, as an electrochemical bioreporter can complete the detection process on-site, it greatly avoids the influence of subjective factors caused by sampling and has high sensitivity and strong specificity.

## Applicability in Soil Contaminant Detection

Soil is a complex matrix that is composed of minerals, organic matter, and organisms. Although the previous study demonstrated that the single bioassay cannot interpret the overall quality of soil, microbial cells exhibited good sensitivity to assess heavy metals and organic pollutants in soil extract (Bierkens et al., 1998). The main obstacle to the application of bioreporter in soil is the opacity of the soil matrix and background luminescence of minerals, which interfere with the visualization of the bioreporter (Rasmussen et al., 2000; Kim et al., 2020). Although bioreporter cells showed good performance in laboratory conditions, the functionality and reliability of bioreporters are difficult to retain under edaphic environments. The insufficient nutrient supply and toxicity of contaminants suppress microbial activities and cause interference (Haft et al., 2009; Wu et al., 2016, 2020b). The direct contact between bioreporter cells with soil particles can enhance the accuracy of bioreporter detection (Magrisso et al., 2009; Jiang et al., 2017), but minerals were found to impact the microbial cell robustness (Cai et al., 2018). For example, iron oxides and manganese oxides have been found to trigger an SOS response and deactivate the bioreporter cells (Cai et al., 2019; Liu et al., 2020).

There are several current strategies to improve the applicability of bioreporters in soil. Soil was mixed with minimal salt solutions to enable visualization of fluorescent and luminescent signals (Song Y. et al., 2014; Yoon et al., 2016d). Soil samples are generally diluted about 5–20 times and vigorously shaken to break down soil aggregates. The resultant mixture and the supernatant can be subject to fluorescence or luminescence measurement. To enhance the adaptability of bioreporter cells in soil, indigenous soil microorganisms, such as *Pseudomonas putida* and *Acinetobacter baylyi*, are preferred to the model strain *E. coli* in bioreporter designs (Zhang et al., 2012).

However, the pretreatment process alters the bioavailability of soil contaminants. The addition of minimal salt solutions solubilizes metals and organics, which generates an overestimated concentration for the bioavailable contaminants (Ivask et al., 2002). The pretreatment of soil samples by sonication or shaking homogenizes and emulsifies hydrophobic organic pollutants (Jiang et al., 2017). The physiochemical properties of the soil environment like pH and moisture vary widely within micrometer distance, which cause the heterogeneous distribution and speciation of contaminants in soil. Previous studies using synchrotron-based X-ray fluorescence spectroscopy found hundred- and thousand-fold differences in concentration of heavy metals like Cu and Ni occurring over micrometer distances (Jacobson et al., 2007; Schmid et al., 2016; Qu et al., 2019). As contaminants heterogeneously distributed at spatial scales relevant to microbes, they trigger a heterogeneous response of bioreporter cells. Single-cell detection is required to assess the spatial heterogeneity of soil contaminants.

To address these issues, attempts have been made to recover the bioreporter cells from the soil matrix with density gradient centrifugation (Hurdebise et al., 2015). Previous studies also functionalized bioreporter cells with magnetic nanoparticles like Fe_3_O_4_, by which bioreporter cells can be isolated under a magnetic field (Jia et al., 2016). Moreover, the immobilization of bioreporter cells on or in solid media enhances their stability in the soil environment, which enables *in situ* measurement. For example, bioreporter cells can be encapsulated into hydrogel beads (Bae et al., 2020). The alginate beads were deployed on the soil surface to detect toluene, and bioluminescence was measured *via* a fiber-optic probe. Moreover, bioreporters can be immobilized on optical fibers, which further simplifies the bioreporter system (Polyak et al., 2001; Kumar et al., 2006). The portability of bioreporter systems requires further research to allow on-site measurements.

## Recent Advances in Soil Contaminant Detection

### Heavy Metals

Early studies have demonstrated metal-sensing bioreporters with a nanomolar detection limit in aqueous samples (Selifonova et al., 1993; Corbisier, 1997). Recent works employed bacteria such as *E. coli, P. putida*, and *A. baylyi* to detect heavy metals in the soil environment (Table 1; Bilal and Iqbal, 2019). Both specific and non-specific metal-sensing bioreporters were constructed to measure Cd, Hg, As, Zn, Cu, Cr, and Pb in soil–water mixture (Table 1). Non-specific bioreporters using the stress-responsive promoter *recA* can respond to a range of heavy metal contaminants based on genotoxicity (Song Y. et al., 2014). The non-specific bioreporter with *recA* as sensing element has a detection limit of 1 mg/kg chromium (Jia et al., 2016). The sensitivity of non-specific bioreporters can be enhanced by using metalloproteins as sensor apparatuses. ZntR is one of the most commonly used non-specific transcriptional regulators, which responds to Zn^2+^, Cd^2+^, and Pb^2+^ (Table 1; Brown et al., 2003). *E. coli* bioreporter with P*_*zntA*_*-*egfp* has a detection limit as low as 0.02 mg/kg for Cd^2+^ in soil samples (Yoon et al., 2016c,d). With the aid of synthetic biology, the selectivity of non-specific regulators can be further enhanced *via* replacing the metal-binding loop. The bioreporters can recognize Cd^2+^ or Hg^2+^ specifically when the metal-binding loop of ZntR was replaced by cadmium- or mercury-binding sequences (Kang et al., 2018). In one recent work, the metal-binding loop (CTTCGCG) was inserted into a loop region of *egfp* to construct a split-protein system (Kim et al., 2019). Upon exposure to Cd and Hg, the binding of metal ions to the metal-binding loop induces a conformational change and restores the fluorescence.

**TABLE 1:** Recent microbial bioreporters designated for heavy metal detection.

**Target analyte**	**Promoter/Reporter construct**	**Reporter**	**Specificity**	**Microbial chassis**	**Application scenarios**	**Detection limit**	**Bioavailability/Chemical availability (%)**	**References**
Cd	P*_*znt*_*/*egfp*-HJ1	Fluorescent	Non-specific	*E. coli*	Extract ^a^	0.95 ± 0.11 mg/kg	–	Kim et al., 2019
	P*_*znt*_*/*egfp*	Fluorescent	Non-specific	*E. coli*	Solution^ b^	0.02 ± 0.032 mg/kg	7.29	Yoon et al., 2016d
	P*_*znt*_*/*egfp*	Fluorescent	Non-specific	*E. coli*	Solution	0.027 ± 0.030 mg/kg	55.15	Yoon et al., 2016c
Hg	P*_*znt*_*/*egfp*-HJ1	Fluorescent	Non-specific	*E. coli*	Extract	0.85 ± 0.12 mg/kg	–	Kim et al., 2019
	P*_*mer*_*/*merR*-*luxCDABE*	Luminescent	Specific	*E. coli*	Extract	0.035 ± 0.15 mg/kg	50.72	Branyikova et al., 2020
	P*_*mer*_/merR*-*luxCDABE*	Luminescent	Specific	*E. coli*	Extract	0.22 μg/L	31.43	Zhang et al., 2016
As	P*_*ars*_/gfp*	Fluorescent	Specific	*E. coli*	Solution	0.119 ± 0.30 mg/kg	12.74	Yoon et al., 2016b
	P*_*ars*_*/*arsR*-*luc*	Luminescent	Specific	*E. coli*	Extract	1.32 ± 0.09 mg/kg	2.84	Hou et al., 2014
	P*_*nik*__*A*_*/*nikR*-*egfp*	Fluorescent	Specific	*E. coli*	Solution	22.0 ± 2.2 μg/kg	11.89	Yoon et al., 2016a
	P*_*ars*_*/*arsR*-*luxAB*	Luminescent	Specific	*E. coli*	Extract	8.06 ± 0.54 mg/kg	66.5	Frick et al., 2019
	P*_*ars*_*/*mCherry*	Fluorescent	Specific	*E. coli*	Solution	0.17 ± 0.033 mg/kg	0.37	Yoon et al., 2016d
Zn	P*_*czcR*__3_*/*egfp*	Fluorescent	Specific	*P. putida*	Extract	7.91 ± 0.22 mg/kg	92.2	Liu et al., 2012
Cu	P*_*cop*_*/*luxAB*	Luminescent	Specific	*P. fluorescens*	Extract	4.34 ± 1.645 mg/kg	99.5	Frick et al., 2019
Cr	P*_*recA*_*/*luxCDABE*	Luminescent	Non-specific	*A. baylyi*	Extract	1 mg/kg	–	Jia et al., 2016
	P*_*recA*_*/*luxCDABE*	Luminescent	Non-specific	*A. baylyi*	Extract	260 mg/kg	–	Jiang et al., 2015
	P*_*recA*_*/*luxCDABE*	Luminescent	Non-specific	*A. baylyi*	Extract	260 mg/kg	–	Jiang et al., 2017
	P*_*rec*__*A*_*/*luxCDABE*	Luminescent	Non-specific	*A. baylyi*	Solution	520 mg/kg	37.1	Song Y. et al., 2014
Pb	P*_*rec*__*A*_*/*luxCDABE*	Luminescent	Non-specific	*A. baylyi*	Solution	2,072 mg/kg	13.0	Song Y. et al., 2014

*^*a*^Extract, the supernatant of soil–water mixture. ^*b*^Solution, soil–water mixture.*

The specific metal-binding regulators can increase both the selectivity and sensitivity of the bioreporter significantly. The metal-sensing repressors like ArsR and MerR function as sensing apparatuses to control the transcription of downstream operons (Kaur et al., 2015; Guo et al., 2019). Selective mercury-sensing system was incorporated in *E. coli via* using regulator protein MerR and promoter *mer* (Branyikova et al., 2020). The detection limit of the mercury-selective bioreporter is 0.035 mg/kg, which is not interfered by other toxic heavy metal ions (Branyikova et al., 2020). The arsenic-specific bioreporter with regulators NikR and ArsR exhibits good sensitivity with a detection limit of 22 μg/kg (Yoon et al., 2016a; Frick et al., 2019).

Due to the toxicity of heavy metal, detoxification genes are fused into regulatory regions to enhance the chassis heavy metal tolerance. For example, after incorporation of the metal transport protein-coding genes, such as *zntA* or *cadA*, microbes can adapt to severely contaminated environments (Biran et al., 2000). Fusion of Pb-binding protein PbrR with the outer membrane protein can allocate heavy metal-binding proteins on the cell surface, which alleviated the toxicity of accumulated Pb in the cell (Wei et al., 2014). On the contrary, deletion of metal exporter genes like *copA* can accumulate heavy metal intracellularly, which enhances the sensitivity of bioreporters with low metal exposure (Kang et al., 2018).

The signal transduction of microbes can be accomplished *via* electrochemical system (Dali et al., 2018). The electrochemical arsenic reporter was constructed in *E. coli* DH5α, which harbors a plasmid carrying *ars* operon promoter-controlled *lacZ* genes. With the presence of arsenate, β-galactosidase transforms p-aminophenyl-β-D-galactopyranoside into p-aminophenol, which can be detected electrochemically outside the cell (Cortes-Salazar et al., 2013). Whole-cell bioreporters can also be incorporated in microbial fuel cells to enable metal measurement. Zn-responsive bioreporter was constructed *via* fusing P*_*zntA*_* promoter with *zntR*, *rib*, and *oprF*. The presence of Zn triggered the riboflavin and porin production, which demonstrated a linear relationship between the maximum voltage with Zn concentration (Khan et al., 2020).

The bioreporters with metalloregulators like MerR and ZntR can detect μg/L of heavy metal ions in the soil–water mixture (Yoon et al., 2016d; Zhang et al., 2016). The corresponding detection limits of bioreporters for heavy metals like As, Hg, and Cd in soil are below mg/kg, which is comparable to that of inductively coupled plasma-atomic emission spectroscopy (ICP-AES) (Martin et al., 1992). The detection limits of whole-cell bioreporters are 10 to 100 times lower than the Chinese regulatory standards and in the same order of magnitude as the US EPA standards (EPA, 2002, 2012; MEE, 2018). Moreover, although the current soil quality standards are established based on chemical analysis, the bioavailable fraction of contaminants determines ecological and toxicological relevance. Previous studies have demonstrated that the bioavailability of heavy metals in soil was only about 10∼50% of chemical-extractable fractions (Table 1). Therefore, the whole-cell bioreporter is sufficiently sensitive and reliable to assess soil heavy metal contamination.

### Organic Pollutants

Different from heavy metals, types of organic contaminants in soil are highly diverse. The first whole-cell bioreporter was constructed by inserting the *lux* operon into a naphthalene catabolic plasmid in *P. fluorescens* (King et al., 1990). The luminescence was induced when exposed to naphthalene or the regulatory inducer metabolite salicylate. Similarly, an alkane bioreporter was constructed using alkane-degrading *A. baylyi* ADP1 carrying transcriptional regulator *alkR*, promoter *alkM*, and reporter *luxCDABE* (Zhang et al., 2012). AlkR recognizes alkanes and activates luminescent gene expression. The bioreporter can stick to the interface of oil and water and emulsify crude oil into micron droplets, which facilitates the detection of the oil spills (Zhang et al., 2012).

Due to the diverse organic contaminants, the contaminant concentration can also be evaluated *via* the central metabolite. For example, most polycyclic aromatic hydrocarbon (PAH) metabolisms occur *via* the salicylate pathway. A whole-cell microbial bioreporter containing *nahAD* and *luxCDABE* gene was constructed to degrade and detect naphthalene (Table 2; Sun et al., 2017). The constitutive promoter P*_*tet*_* is fused to catabolic gene *nahAD* to enhance the degradation of naphthalene to salicylate. Salicylate binds to the SalR regulator and activates the P*_*sal*_* promoter, which triggers the expression of *salAR* operon and *luxCDABE* gene (Sun et al., 2017). The bioreporter targeting salicylate exhibits good sensitivity, with a detection limit of 0.018 mg/kg. As bioreporter construction for different PAH molecules is laborious, quantification of the parent molecules *via* detection of metabolic intermediates is a feasible approach.

**TABLE 2:** Current microbial bioreporters designated for the detection of organic pollutants (PAHs, PCBs, pesticides, antibiotics).

**Target analyte**	**Reporter construct**	**Reporter**	**Specificity**	**Microbial chassis**	**Application scenarios**	**Detection limit**	**Bioavailability/Chemical availability (%)**	**References**
Alkanes	P*_*alkM*_*/*alkR-luxCDABE*	Luminescent	Specific	*A. baylyi*	Extract	0.1 mg/kg	–	Zhang et al., 2012
Benzo[a]pyrene	P*_*rec*__*A*_*/*luxCDABE*	Luminescent	Non-specific	*A. baylyi*	Solution	0.5 mg/kg	9.4	Song Y. et al., 2014
Toluene	P*_*grp*__*E*_*/*luxCDABE*	Luminescent	Non-specific	*E. coli*	Soil	2 mg/g	–	Bae et al., 2020
	Pu/*xylR-luxCDABE*	Luminescent	Specific	*A. baylyi*	Extract	13.8 mg/kg	–	Wang Y. et al., 2014
Polychlorinated Biphenyls	P_*m*_/*gfpmut3b*	Fluorescent	Specific	*P. fluorescens*	Soil	0.1 mg/kg	–	Liu et al., 2010
Phenanthrene	P*_*lac*_*/*luxCDABE*	Luminescent	Non-specific	*E. coli*	Extract	2 mg/kg	–	Gu and Chang, 2001
Naphthalene	P*_*sal*_*/*salR-luxCDABE*	Luminescent	Non-specific	*A. baylyi*	Solution	0.018 mg/kg	–	Sun et al., 2017
Mitomycin C	P*_*rec*__*A*_*/*luxCDABE*	Luminescent	Non-specific	*A. baylyi*	Extract	8.36 μg/kg	–	
	P*_*rec*__*A*_*/*luxCDABE*	Luminescent	Non-specific	*A. baylyi*	Solution	0.4 mg/kg	65.8	Song Y. et al., 2014
Tetracycline	*tet*/*mCherry*	Fluorescent	Specific	*E. coli*	Extract	5.32 μg/kg	89.5	Bae et al., 2020

Advances in cell immobilization are beneficial for *in situ* measurement. In one recent study, bioreporter cells are encapsulated into alginate beads to enhance the stability of bioreporters in the soil environment (Bae et al., 2020). The bioreporter can sense toluene *via* stress-responsive promoter P*_*grpE*_*. The alginate beads were deployed on the surface of the soil to detect volatile contaminants like toluene, and the bioluminescence was measured *via* a fiber-optic probe. Bioelectrochemical systems can also be employed in organic contaminant detection, in which bioreporters were immobilized on electrodes (Boron et al., 2020). Recently, Boron et al. (2020) have developed a herbicide bioreporter based on the suppression of photosynthesis by atrazine. In this system, microalgae *Monoraphidium contortum* are fixed on the screen-printed graphite electrode. The decreased oxygen production can be measured by electrochemical methods (Boron et al., 2020).

Antibiotic residues in soil are emerging contaminants that impact soil microbial community and cause the accumulation of antibiotic resistance genes. Whole-cell microbial bioreporters can be constructed by fusing an antibiotic-sensitive promoter or an inducible gene promoter with a reporter gene (Parthasarathy et al., 2018; Ma et al., 2020). The previous whole-cell bioreporters were mainly used to detect tetracyclines in aqueous samples (Korpela et al., 1998; Kurittu et al., 2000). A recent tetracycline fluorescent bioreporter was constructed to analyze soil samples using *tetRO* tetracycline-responsive control region and *gfp* or *mCherry* reporter gene, which had a detection limit of 5.32 μg/kg soil (Bae et al., 2020). Using 96-well microplates, this fluorescent bioreporter method can measure 96 or more samples simultaneously within 6 h.

Previous studies have demonstrated that the sensitivity of bioreporters toward petroleum contaminants in aqueous samples has been improved down to the ppm and ppb range, approaching the sensitivities of chemical analysis like GC-MS (Jiang et al., 2020). The high sensitivity was retained in the soil–water mixture (Table 2; Zhang et al., 2012; Sun et al., 2017). Therefore, the bioreporter is sensitive enough to evaluate whether the concentration of contaminants like naphthalene exceeds the ppm-level regulatory standards (EPA, 2006; Kuppusamy et al., 2019). Meanwhile, the sensitivity still needs to be improved to evaluate organic pollutants (e.g., benzo[a]pyrene) with a sub-ppm regulatory limit.

## Future Prospects

Whole-cell microbial bioreporters have exhibited promising performance in soil spiked with a single contaminant. But it is still challenging to differentiate multiple contaminants with a wide concentration range. The emergence of novel contaminants in soil also triggers requirements to construct new bioreporter modules. With the increasing capacity of high-throughput genomic sequencing, metagenome mining and synthetic biology provide new opportunities to explore new transcription factors or operators to improve the sensing modules (Jiang et al., 2016; Ho et al., 2018; Hicks et al., 2019). The development of novel genetic logic gates has the potential to efficiently modulate and transfer cellular signals to enhance the sensitivity and specificity while avoiding false-positive signals (Bansal et al., 2010). Applying alternative microbial chassis like basophils and halophiles enables the analysis in an extremely unfriendly environment.

In addition, significant efforts need to be undertaken to improve the measurement processes. Most previous contamination measurements were involved in soil solution or extract preparation. However, the pretreatment process alters the bioavailability of soil contaminants. For example, sonication of soil samples homogenizes and emulsifies hydrophobic organic pollutants (Jiang et al., 2017). Removal of soil particles from extracts compromised the accuracy of bioreporter detection (Magrisso et al., 2009; Jiang et al., 2017). One way to resolve this problem is to avoid the dilution/extraction step. Therefore, *in situ* detection based on bioelectrochemical systems and optic techniques will be necessary to advance the assessment of the actual contamination status, which soil bacteria are exposed to.

*In situ* measurement or monitoring in the field is difficult, which bears a risk to release genetically modified microorganisms. Different strategies have been attempted to envelop bacteria or fix microbial cells on the bottom of a microtiter plate using agar or alginate (Bjerketorp et al., 2006; Odaci et al., 2009; Ferro et al., 2012; Bae et al., 2020). Nanomaterials can be a promising alternative to further improve cell immobilization and signal transduction. For example, graphene-based materials have a large specific surface area with excellent thermal and electrical conductivity, which can adsorb organic contaminants for signal amplification (Zhang et al., 2010; Cho et al., 2020; Wu et al., 2020a). These unique properties make graphene-based electrodes a good candidate to immobilize bioreporters and transduce electrical signals (Li et al., 2017). The electrochemistry of target contaminants and electrochemically active microorganisms can further improve the selectivity and sensitivity.

The portability of bioreporter systems requires to be increased to complete all the analyses and data interpretation in the field. The flexibility of the bioreporter permits its easy adaptation to miniaturized and autonomous devices. For example, the visual signals from bioreporters in microfluidic devices can be captured by the miniaturized photomultiplier detectors (Roggo and van der Meer, 2017). The electrochemical bioreporter can be easily integrated into field-applicable electric devices (Ben-Yoav et al., 2009; Boron et al., 2020). The integration of emerging disciplines such as artificial intelligence, big data, smart mobile devices, and drones will be necessary to close the gap between research and field application and realize long-term online monitoring of soil contamination.

## Author Contributions

NZ and YW reviewed the literature, designed the concept, wrote the manuscript, and prepared the figures. WC, QH, and PC revised the manuscript. All authors approved the manuscript for publication.

## Conflict of Interest

The handling editor declared a past co-authorship with one of the authors YW. The remaining authors declare that the research was conducted in the absence of any commercial or financial relationships that could be construed as a potential conflict of interest.
